# CircSMARCA5: A key circular RNA in various human diseases

**DOI:** 10.3389/fgene.2022.921306

**Published:** 2022-08-23

**Authors:** Yi Zhu, Gaozhen Huang, Shihao Li, Hong Xiong, Ruiqi Chen, Ling Zuo, Hongwei Liu

**Affiliations:** ^1^ Department of Urology, Affiliated Hospital of Guangdong Medical University, Zhanjiang, China; ^2^ Department of Traditional Chinese Medicine, The Second Affiliated Hospital of Guangdong Medical University, Zhanjiang, China

**Keywords:** circular RNA, cancer, circSMARCA5, miRNA sponge, biomarker

## Abstract

Circular RNAs (circRNAs) are recognized as a novel type of single-stranded endogenous noncoding RNA molecule with the characteristics of tissue specificity, sequence conservation and structural stability. Accumulating studies have shown that circRNAs play a unique biological role in different kinds of diseases. CircRNAs can affect tumor proliferation, migration, metastasis and other behaviors by modulating the expression of downstream genes. CircSMARCA5, an example of a circRNA, is dysregulated in various noninfectious diseases, such as tumors, osteoporosis, atherosclerosis and coronary heart disease. Furthermore, recent studies have demonstrated that circSMARCA5 is associated with the occurrence and development of a variety of tumors, including gastric cancer, glioblastoma, hepatocellular carcinoma, multiple myeloma, colorectal cancer, breast cancer and osteosarcoma. Mechanistically, circSMARCA5 primarily acts as a sponge of miRNAs to regulate the expression of downstream genes, and can serve as a potential biomarker for the diagnosis of malignant tumors. This review summarizes the biological roles of circSMARCA5 and its molecular mechanism of action in various diseases. Moreover, the meta-analysis of some publications showed that the expression of circSMARCA5 was significantly correlated with the prognosis of patients and tumor TNM stage, showing that circSMARCA5 has the potential to be a prognostic marker.

## 1 Introduction

In the past few decades, although great progress has been made in the treatment of noncommunicable diseases, there are still multiple diseases such as tumors and cardiovascular diseases with limited treatment options. For example, in 2020, it was reported that approximately 19.3 million new cases of cancer were diagnosed and nearly 10 million cancer deaths occurred (Sung et al., 2021). The number of deaths caused by cancers is increasing rapidly (Siegel and Jemal 2020) and new treatment approaches need to be developed to increase the cure rates.

**FIGURE 1:**
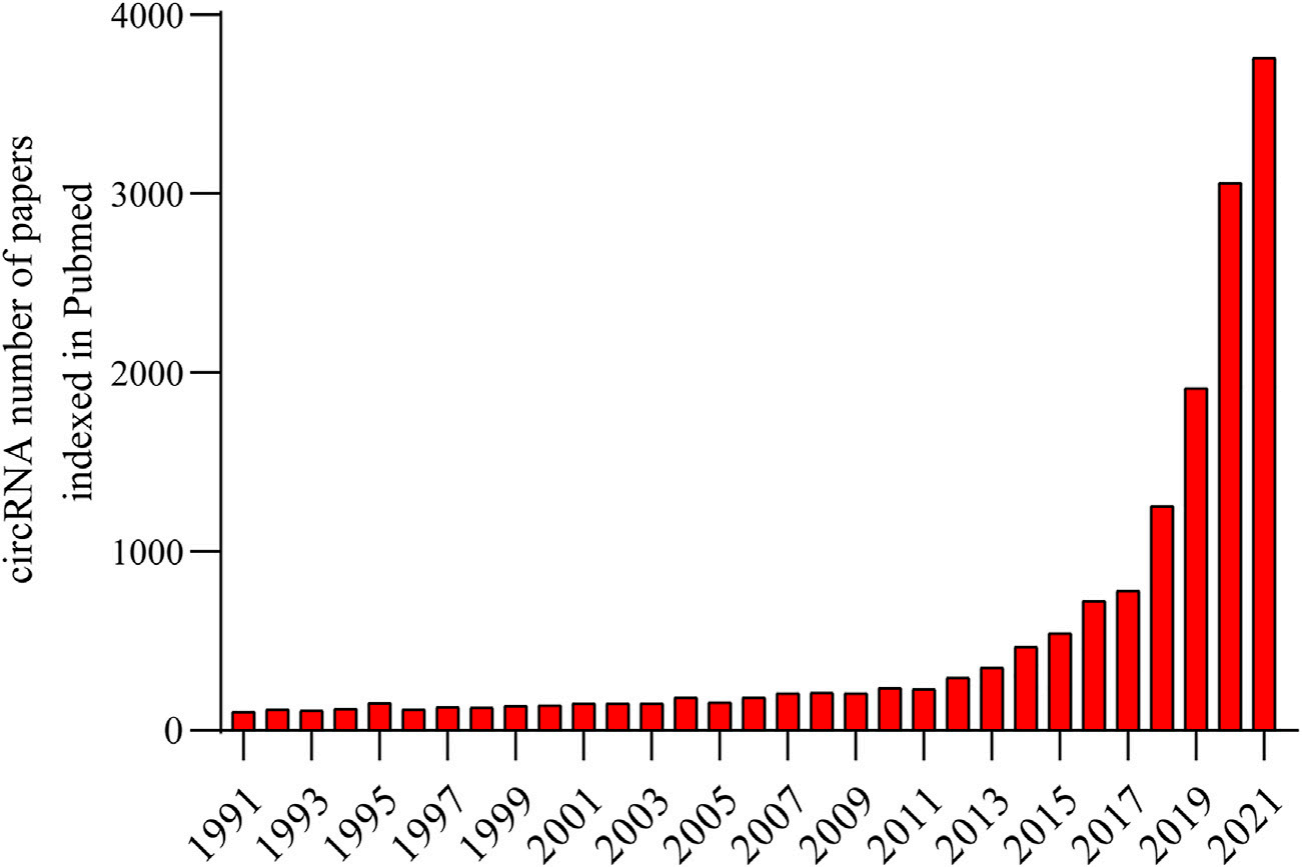
The number of circRNA publications published annually has grown dramatically exponentially in PubMed from 1991 to 2021, especially in the past 5 years.

In recent years, many noncoding RNAs (ncRNAs), including long noncoding RNAs, miRNAs and circular RNAs (circRNAs), have been found to play vital roles in various diseases and have the potential to become therapeutic targets for diseases (Beermann and Viereck 2016; Zhang and Xiao, 2018). CircRNAs have attracted much attention due to their special roles in diseases and this rising interest is reflected in Figure 1. Among numerous circRNAs, circSMARCA5 is aberrantly expressed in a variety of diseases. This review summarizes the expression of circSMARCA5 and its related molecular pathogenesis in various diseases. Furthermore, a meta-analysis was conducted to summarize the prognostic value of circSMARCA5 in malignant tumors.

## 2 Formation of CircRNAs

**FIGURE 2:**
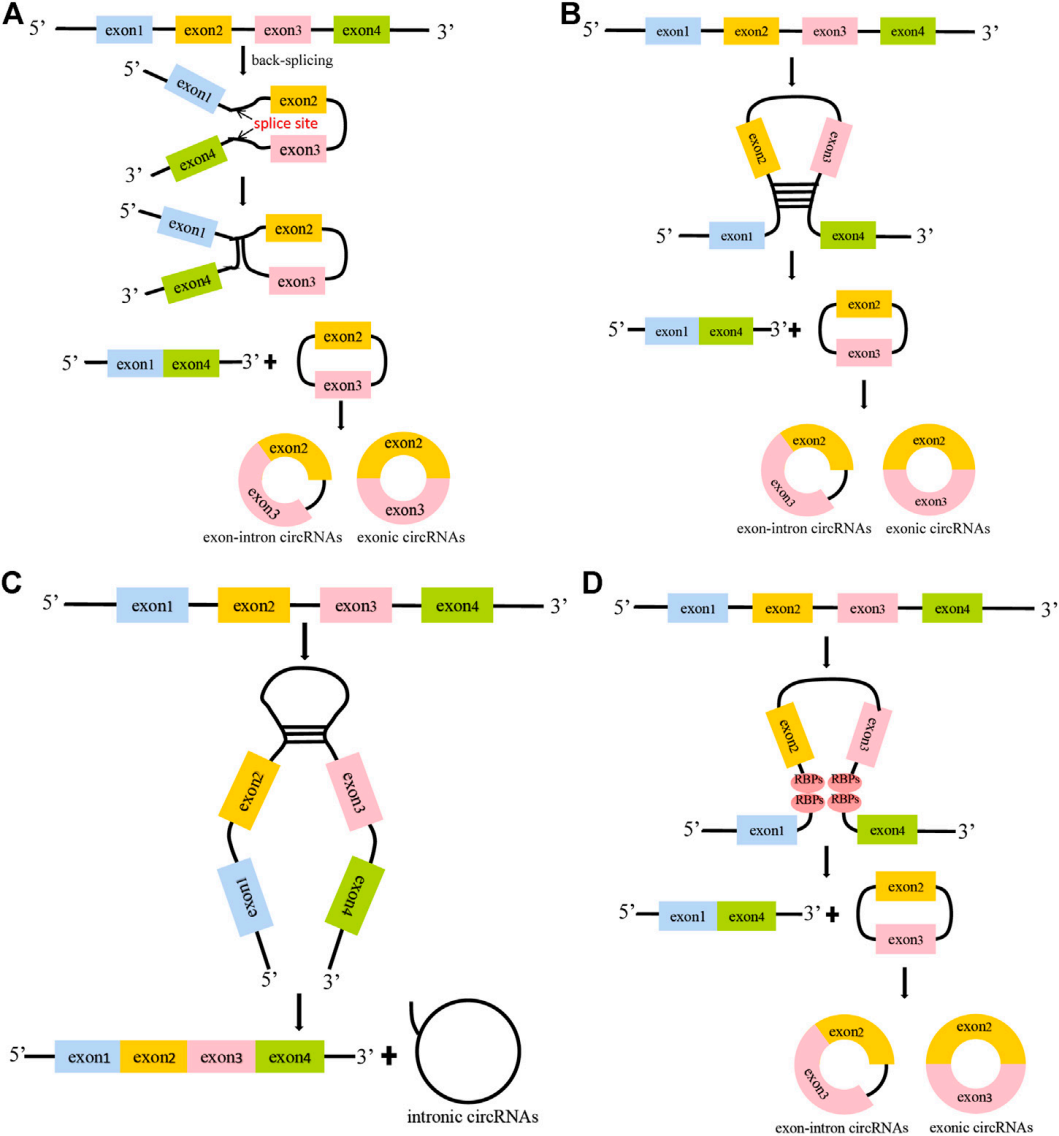
The formation mechanisms of circular RNAs. **(A)**. Lariat-driven circularization. The splice donor of the downstream exon of the pre-mRNA binds to the splice acceptor of the upstream exon, thereby forming exon-intron circRNAs. When the introns are removed, the exons are linked by 3–5′ phosphodiester bonds to form exonic circRNAs. **(B–C)**. Intron pair-driven circularization. The introns flanking the exons of the pre-mRNA are base -paired through ALU repeats to generate exon-intron circRNAs, exonic circRNAs and intronic circRNAs. **(D)**. RBPs-mediated circularization. RBPs bind to RBP binding sites in intron sequences, and the interaction promotes the production of circRNAs.

CircRNAs, a type of noncoding RNA, were discovered in the 1970s and originally considered to be byproducts of RNA molecular mis-splicing (Patop and Kadener 2019; Sanger, 1976). It was not until the past decade that people realized the underlying importance of circRNAs in diseases. There are three generally accepted circRNA circularization mechanisms, including lariat-driven circularization, intron pair-driven circularization and RNA binding proteins (RBPs)-mediated circularization (Jeck et al., 2013; Vicens, 2014; Yang G et al., 2021) (Figure 2). In lariat-driven circularization, a lariat structure is formed by the covalent binding of the splice donor of the downstream exon of the precursor mRNA (pre-mRNA) and the splice acceptor of the upstream exon, leading to the formation of exon–intron circRNAs (EIciRNAs) and exonic circRNAs (Figure 2A). In the mechanism of intron pair-driven circularization, circularization is generated from the introns flanking the exons of the pre-mRNA sequence, shaping a circular structure through the base pairing of the ALU repeat sequence (Figures 2B,C). In addition, it has been reported that the biogenesis of circRNAs is regulated by RBPs (Conn et al., 2015; Dong et al., 2019a). In RBPs-mediated circularization, a circular structure is generated by the connection of RBPs to introns on both flanking exons of the pre-mRNA sequence (Figure 2D). CircRNAs are primarily categorized into three types based on their source, namely, exonic circRNAs, intronic circRNAs and EIciRNAs, and most circRNAs are present in the cytoplasm. Despite the existence of these different kinds of circRNAs, most of the circRNAs studied thus far are generated by back-splicing of exons from the pre-mRNA (Li and Chen 2018). Due to the lack of terminal 5′ caps and 3′ poly(A) tails, circRNAs are more resistant to the degradation of RNase R than linear mRNA and could be highly stable in the cytoplasm (Liu et al., 2018; Dong et al., 2019b). CircRNAs are widely present in tissues, blood and urine, and have the characteristics of tissue specificity, sequence conservation and structural stability (Memczak et al., 2013; Yang G et al., 2021).

## 3 Biological functions of CircRNAs

**FIGURE 3:**
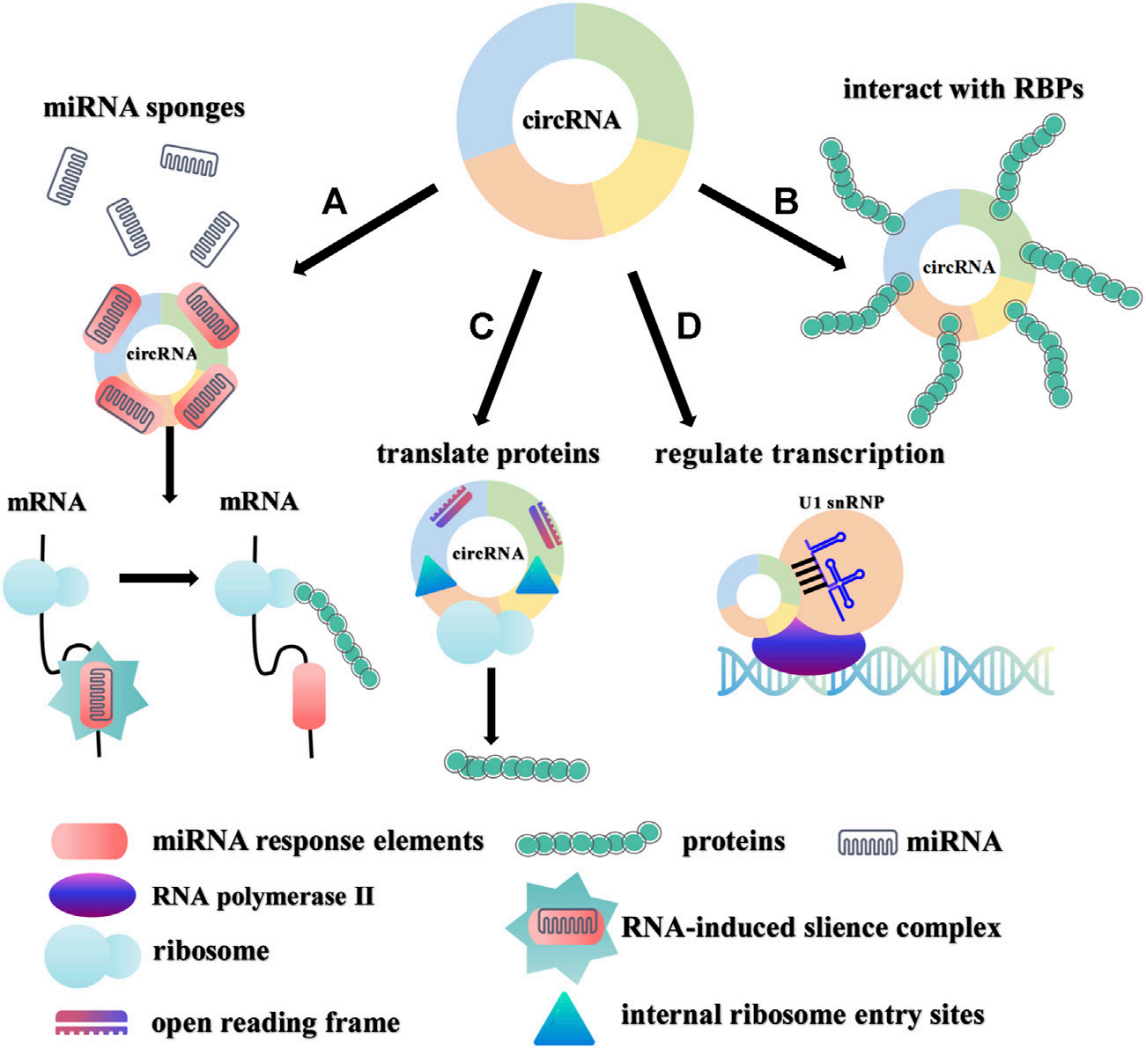
Biological functions of circRNAs. **(A)**. miRNA sponges. circRNAs can function as miRNA sponges to regulate the expression of target genes. **(B)**. Interaction with RBPs. circRNAs can interact with RBPs to affect downstream signals. **(C)**. Protein coding. circRNAs with IRESs and ORFs can translate certain proteins/peptides to perform special functions. **(D)**. Transcriptional regulation. EIciRNAs and U1 small nuclear ribonucleic proteins (U1 snRNPs) are co-localized to the promoter of the parental gene and promote transcription of the parental gene with the participation of RNA polymerase II.

CircRNAs can play major roles in the progression of various diseases through their various biological effects. CircRNAs exert their biological functions by serving as sponges of miRNA (Hansen, 2013; Memczak et al., 2013), interacting with RBPs (Huang Z et al., 2020), participating in protein coding (Pamudurti et al., 2017) and regulating transcription (Li et al., 2015) (Figure 3). Many circRNAs with miRNA response elements have been discovered to play essential biological roles by acting as competitive endogenous RNAs (Hansen, 2013). For example, ciRS7 (also called CDR1as), a typical sponge of miR-7, contains more than 70 miR-7 binding sites (Hansen, 2013; Memczak et al., 2013). CircSPARC upregulates the expression of JAK2 by competitively binding to miR-485-3p, and ultimately enhances the migration and invasion of colorectal cancer (Wang et al., 2021). In addition, circRNAs have also been discovered to perform biological functions by interacting with RBPs or participating in protein coding (Huang Q et al., 2020; Lei et al., 2020; Prats et al., 2020). As an example, the combination of circLIFR and MutS homolog 2 protein improved the chemotherapy sensitivity of bladder cancer through the MutSα/atm-p73 axis (Zhang X et al., 2021). Furthermore, some studies have also elucidated that circRNAs that rely on the internal ribosome entry sites (IRESs) and open reading frames (ORFs) initiate the protein translation process independent of the 5′ cap (Yang and Wang, 2019). For instance, circMAPK1 inhibits the progression of gastric cancer by encoding the protein MAPK1-109aa to inhibit downstream pathway activation (Jiang et al., 2021). Finally, circRNAs also regulate transcription to affect the expression of their parental genes (Li et al., 2015; Xu et al., 2020). Increasing evidence suggests that dysregulated circRNAs are related to the occurrence and development of various diseases and have the potential to become targets for disease therapy.

## 4 Results of the publication search

**FIGURE 4:**
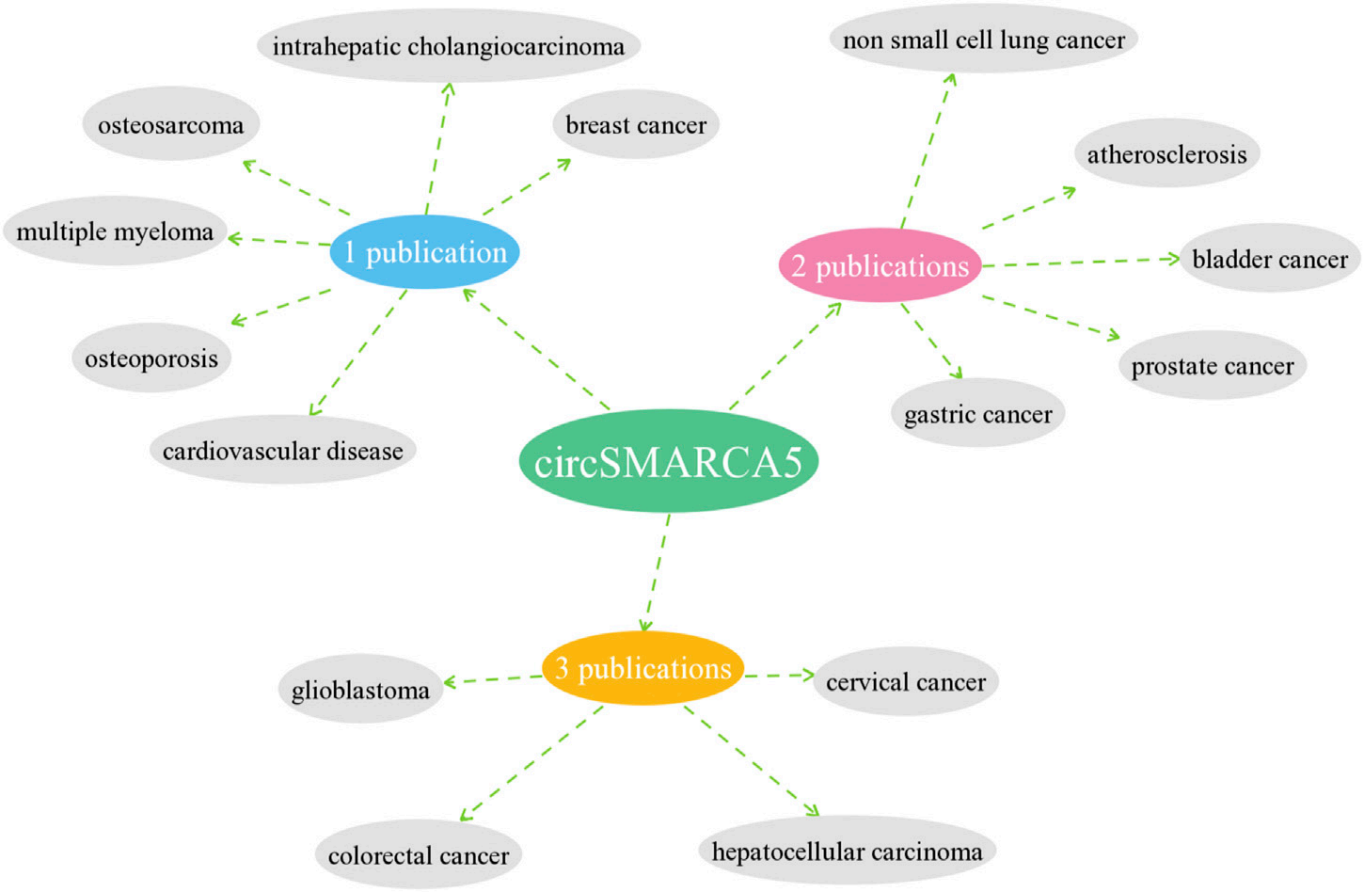
Descriptive statistics of the number of studies of circSMARCA5 in each disease.

**FIGURE 5:**
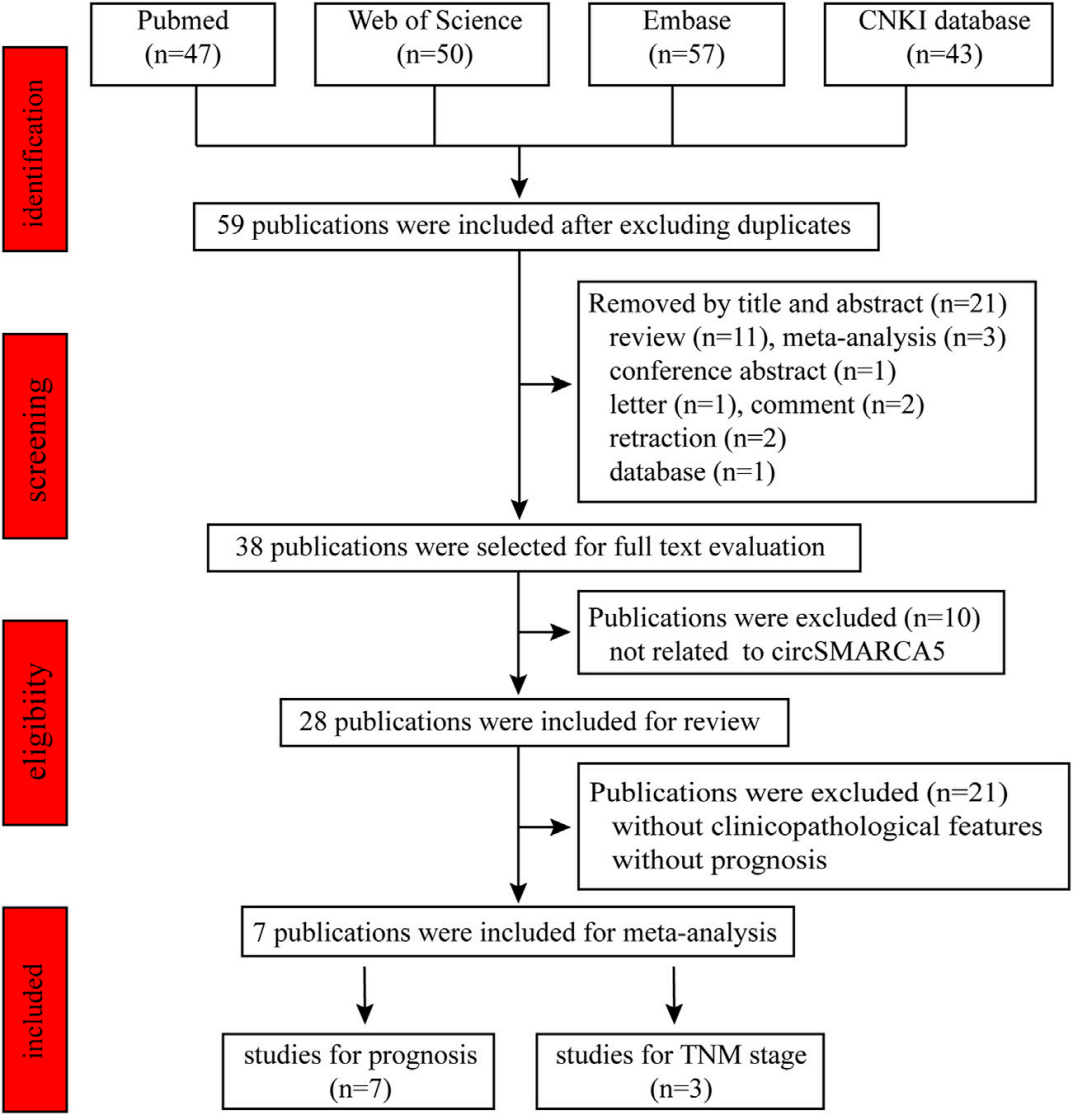
A flow chart of screening eligible articles for meta-analysis.

We retrieved 197 publications from Pubmed, CNKI, Web of Science and Embase database using the keywords circSMARCA5, circular RNA SMARCA5, circular RNA cSMARCA5 or hsa_circ_0001445. Fifty-nine publications about circSMARCA5 were enrolled after excluding the duplicates. By reviewing title and abstract, we excluded 21 publications, including 11 reviews, 3 meta-analyses, 1 conference abstract, 1 letter, 2 comments, 2 retractions and 1 database. Then, we reviewed full text and 10 publications not related to circSMARCA5 were excluded. Finally, 28 original research papers were included in the scope of the review (Figure 4) and 7 publications were enrolled for meta-analysis (Figure 5).

## 5 Formation, characteristics and expression of circSMARCA5

**FIGURE 6:**
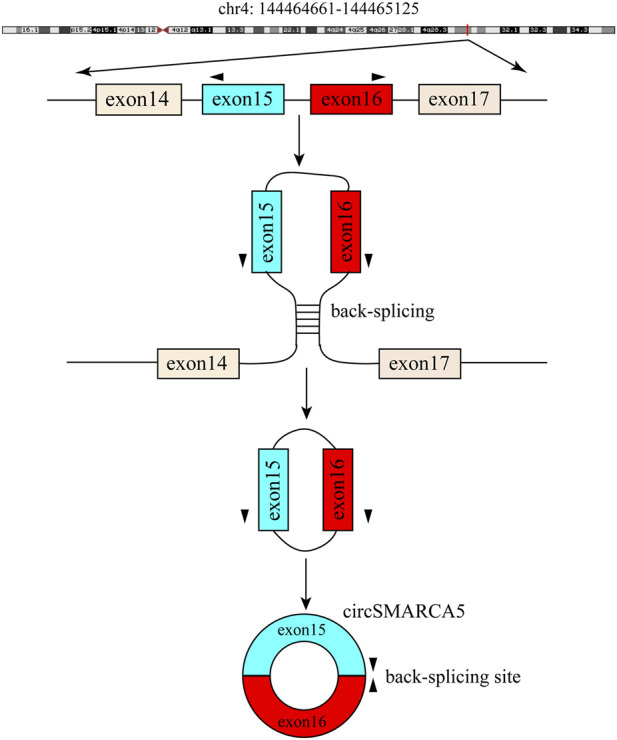
Generation of circSMARCA5. Exons 15 and 16 of SMARCA5 are back-spliced to form circSMARCA5.

CircSMARCA5 (circbase ID: hsa_circ_0001445), which is generated from the back-splicing of exons 15 and 16 of SMARCA5 (*Homo sapiens* SWI/SNF related, matrix associated, actin dependent regulator of chromatin, subfamily a, member 5) (Figure 6). The NCBI database shows that SMARCA5 (GenBank Accession ID NM_003601) is located on chromosome 4q31.21 and comprises 7,684 base pairs. Several studies have shown that circSMARCA5 mainly exists in the cytoplasm (Han et al., 2021). Moreover, it was reported that the half-life of circSMARCA5 surpassed 24 h, while the corresponding linear SMARCA5 mRNA’s half-life was only 4 h (Yu et al., 2018; Cai and Zuo, 2019), showing that circSMARCA5 could stably exist in the cytoplasm. Moreover, circSMARCA5 is resistant to RNase R compared with SMARCA5 mRNA (Yu et al., 2018; Xu et al., 2020). The circSMARCA5 expression level is disordered in different cancers (Table 1) and it can act as a potential prognostic biomarker for liver cancer, gastric cancer, glioblastoma, multiple myeloma and colorectal cancer. Moreover, the expression level of circSMARCA5 is correlated with clinicopathological parameters of different cancers (Table 2).

**TABLE 1:** The expression and roles of circSMARCA5 in various cancers.

Tumor types	Samples	Expression	Mechanism	Functional roles	Ref
GC	23 pairs	down	miR-346/FBXL2	suppress proliferation, migration and invasion	Li and Hu (2019)
	60 pairs	down	—	inhibit proliferation, migration, and invasion	Cai and Zuo, (2019)
GBM	56 tumor 7 normal	down	SRSF1/SRSF3/PTBP1/PTBP2	inhibit migration and angiogenesis	Barbagallo et al. (2018)
HCC	133 pairs	down	—	inhibit proliferation, invasion and metastasis, promote late apoptosis	Li and Hu (2019)
	73 pairs	down	—	suppress proliferation, migration and invasion, enhance apoptosis	Zhang and Xiao, (2018)
	40 pairs	down	DHX9/circSMARCA5/miR-17-3p/miR-181b-5p/TIMP3	suppress growth, migration and distant metastasis	Yu et al. (2018)
NSCLC	66 pairs	down	miR-670-5p/RBM24	suppress growth	Zhang et al. (2020)
	460 pairs	down	—	inhibit proliferation and strengthen hemotherapy sensitivity	Tong, (2020)
ICC	92 pairs	down	—	inhibit proliferation and increase chemotherapy sensitivity	Lu, (2020)
MM	105 tumor, 36 normal	down	miR-767-5p	inhibit proliferation and promote apoptosis	Liu et al. (2019)
CRC	35 pairs	down	miR-552/Wnt /YAP1	suppress proliferation, migration, invasion and promote apoptosis	Yang G et al. (2021)
	45 pairs	down	miR-39-3p/ARID4B	inhibit proliferation, migration and invasion	Miao et al. (2020)
CC	30 pairs	down	miR-620	inhibit proliferation, migration and invasion	Tian, (2018)
	20 pairs	down	SND1/YWHAB	suppress proliferation and invasion, enhance apoptosis	Zhang Z et al. (2021)
BC	—	down	SMARCA5	suppress DNA repair capacity and improve chemotherapy sensitivity	Xu et al. (2020)
PCa	30 pairs	up	miR-432/PDCD10	promote proliferation, metastasis, and glycolysis	Dong et al. (2021)
	21 pairs	up	—	promote proliferation, inhibit apoptosis and affect cell cycle	Kong et al. (2017)
BCa	156 tumor 71 normal	up	—	promotes proliferation, migration, invasion and inhibit apoptosis	Tan and Liang. (2019)

Abbreviations: GC: gastric cancer; GBM: glioblastoma; HCC: hepatocellular carcinoma; NSCLC: non-small -cell lung cancer; ICC: intrahepatic cholangiocarcinoma; MM: multiple myeloma; CRC: colorectal cancer; CC: cervical cancer; BC: breast cancer; PCa: prostate cancer; BCa: bladder cancer.

**TABLE 2:** The clinicopathological features of circSMARCA5 in several human cancers.

Tumor types	Clinicopathological features	Ref
GC	poor tumor differentiation, more lymph node metastasis, vascular invasion, high AJCC stage, poor OS and DFS	Cai and Zuo, (2019)
GBM	advanced histological grade, more blood vascular microvessel density, poor OS and PFS	(Barbagallo et al., 2018; Barbagallo et al., 2019)
HCC	poor tumor differentiation, high TNM stage, microvascular invasion, large tumor size and more tumor lesions	(Yu et al., 2018; Zhang et al., 2018; Li ad Hu, 2019)
NSCLC	large tumor size, more lymphatic metastasis and high TNM stage	(Tong, 2020; Zhang et al., 2020)
ICC	ECOG performance score, advanced TNM stage and abnormal CA199 status	Lu, (2020)
MM	high beta-2-microglobulin levels, poor complete response and short OS and PFS after effective chemotherapy	Liu et al. (2019)
BCa	larger tumor size, higher tumor stage, lymphatic metastasis and worse DFS and OS	(Tan and Liang. 2019; Zhang X et al., 2021)

Abbreviations: GC: gastric cancer; GBM: glioblastoma; HCC: hepatocellular carcinoma; NSCLC: non-small -cell lung cancer; ICC: intrahepatic cholangiocarcinoma; MM: multiple myeloma; BCa: bladder cancer.

## 6 Mechanism of action of circSMARCA5

**FIGURE 7:**
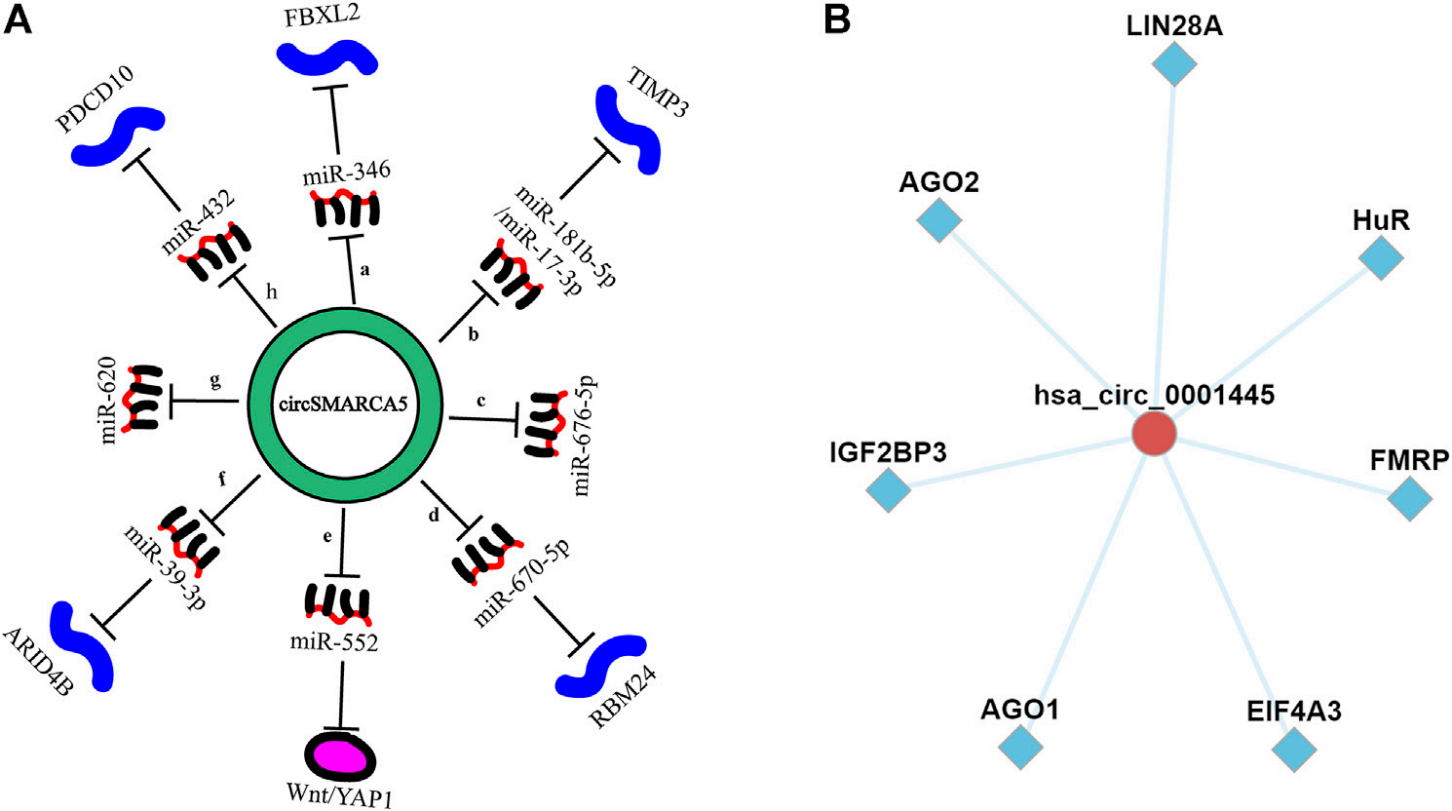
**(A)**. CircSMARCA5 acts as miRNA sponges in various cancers. (a). gastric cancer; (b). hepatocellular carcinoma; (c). non-small -cell lung cancer; (d). multiple myeloma; (e,f). colorectal cancer; (g). cervical cancer; (h). prostate cancer. **(B)**. CircFunBase database was used to predict potential RNA-binding proteins that can bind to circSMARCA5.

CircSMARCA5 plays a regulatory role by serving as a competitive endogenous RNA (Figure 7A), binding to its parental genes (Xu et al., 2020) or interacting with RBPs. A study showed that the sequence of circSMARCA5 contains a binding site (GAUGAA) capable of binding to the SRSF1 protein (Barbagallo et al., 2021). Moreover, circSMARCA5 can also bind to RNA-binding protein SND1 (an oncoprotein), thereby inhibiting the proliferation, invasion and metastasis of cervical cancer (Zhang Z et al., 2021). Given that there are few studies on circSMARCA5 and RNA-binding proteins in the existing literature, we used CircFunBase online database (http://bis.zju.edu.cn/CircFunBase/) to predict potential RNA-binding proteins that can bind to circSMARCA5. Results showed that multiple RNA-binding proteins, including AGO2, IGF2BP3, AGO3, EIF4A3, FMRP, HuR and LIN28A proteins, have the potential to bind to circSMARCA5 and may participate in RNA-protein interactions (Huang Z et al., 2020) (Figure 7B). In addition, by analyzing the sequence of circSMARCA5 in CircBank (http://www.circbank.cn/index.html) and SRAMP (http://www.cuilab.cn/sramp) database, we found that the sequence of circSMARCA5 has ORFs, IRESs and three N6-methyladenosine modification sites, indicating that circSMARCA5 has the potential to code protein or functional polypeptide.

## 7 CircSMARCA5 and tumors

### 7.1 CircSMARCA5 and gastric cancer

Globally, gastric cancer (GC) is a common disease of the digestive system (Sung et al., 2021), and it is responsible for more than one million new patients annually. *Helicobacter pylori* infection is the most important risk factor for GC, and a poor diet can also lead to GC (Smyth et al., 2020). Therefore, treating *Helicobacter pylori* infection and improving the diet are effective measures to prevent GC. In the last few years, the role of circRNAs in GC has attracted increasing amounts of attention (Shan et al., 2019; Li et al., 2020). Numerous circRNAs were discovered to be dysregulated in GC and involved in the occurrence and development of GC (Ding et al., 2019; Huang et al., 2019; Jie et al., 2020; Luo et al., 2020; Peng et al., 2020). Recently, circSMARCA5 has been found to play an antitumor role in GC.

Cai et al. evaluated circSMARCA5 levels in 60 pairs of GC tissues and matched paracancerous tissues and the circSMARCA5 levels were remarkably reduced in cancer tissues compared to paired normal tissues (Cai and Zuo, 2019). These findings were similar to those of Li et al. (Li and Hu, 2019) Similarly, the plasma circSMARCA5 expression level in GC patients was also reduced compared with that in healthy controls. Moreover, circSMARCA5 in plasma remained stable under some harsh conditions (Cai and Zuo, 2019). Functional experiments were also performed, showing that circSMARCA5 overexpression notably suppressed GC cell proliferation, migration, and invasion (Cai and Zuo, 2019; Li and Hu, 2019).

To assess the diagnostic utility of circSMARCA5 in GC, a receiver operating characteristic (ROC) curve was applied to discriminate GC patients and healthy controls, and the area under the ROC curve (AUC) value was 0.806, which meant that circSMARCA5 has a certain diagnostic utility for GC. Furthermore, the clinicopathological features of GC, such as the degree of tumor differentiation, lymph node metastasis, vascular invasion and AJCC stage, were negatively associated with the level of circSMARCA5 (Cai and Zuo, 2019). Mechanistically, circSMARCA5 inhibits GC progression by serving as a sponge of miR-346 to upregulate FBXL2 expression (Li and Hu 2019). Kaplan-Meier analysis demonstrated that patients with low circSMARCA5 expression had worse overall survival (OS) and disease-free survival (DFS). Multivariate Cox regression analysis also showed that circSMARCA5 levels were an independent prognostic factor for the OS and DFS of GC patients (Cai and Zuo, 2019). In summary, circSAMRCA5 can be regarded as a promising marker for the prognosis and diagnosis of GC.

### 7.2 CircSMARCA5 and glioblastoma

Glioblastoma (GBM) is a highly malignant tumor of the brain and central nervous system. Approximately 12,000 GBM patients are diagnosed in the United States every year (Ostrom et al., 2020), and the 5-years OS rate is only 6.8% (Wen G et al., 2020). Emerging evidence has suggested that circRNAs play a critical role in the progression of GBM and are promising therapeutic targets for the treatment of GBM (Yang et al., 2018; Xia et al., 2019; Zhang et al., 2019; Lou et al., 2020; Wu et al., 2021). Recently, it was reported that circSMARCA5 was associated with the occurrence and development of GBM, and its functions and detailed mechanisms are as follows.

Barbagallo et al. found that the levels of circSMARCA5 in GBM tissues were decreased compared to those in normal tissues (Barbagallo et al., 2018). Furthermore, the grades of GBM were negatively correlated with the abundance of circSMARCA5. *In vitro* experiments showed that circSMARCA5 overexpression suppressed the GBM cell migration rate but did not affect the viability of the cells. Mechanistically, circSMARCA5 interacted with RBP serine and arginine rich splicing factor 1 (SRSF1), an oncoprotein associated with cell migration, and suppressed its expression. This inhibition led to an increase in the expression of the downstream SRSF3 EX4 subtype, and the SRSF3 EX4 subtype interacted with two other splicing factors, polypyrimidine tract binding protein 1 (PTBP1) and PTBP2, thereby significantly suppressing the migration of GBM cells (Barbagallo et al., 2018; Barbagallo et al., 2021). In addition, Barbagallo et al. (Barbagallo et al., 2019) also found that the abundance of circSMARCA5 was negatively correlated with proangiogenic VEGFA mRNA isoform expression and blood vascular microvessel density (MVD), which meant that low expression of circSMARCA5 might promote more angiogenesis in GBM. Moreover, patients with low expression of circSMARCA5 had shorter OS and progression-free survival (PFS). In another study, Han et al. (Han, 2021) demonstrated that the level of circSMARCA5 was remarkably upregulated after treatment with exosomes derived from GBM cells. Furthermore, circSMARCA5 expression induced by exosomes promoted the proliferation, migration, and invasion of GBM cells. Mechanistically, circSMARCA5 increased SNX5 levels by serving as a sponge of miRNA-127-5p, thereby enhancing the progression of GBM.

### 7.3 CircSMARCA5 and hepatocellular carcinoma

Hepatocellular carcinoma (HCC) is a malignant tumor originating from liver cells with an increasing incidence (Chidambaranathan-Reghupaty., 2021; Sung et al., 2021). In 2020, there were 42,810 cases diagnosed and 30,160 cases died of HCC in the United States (Siegel and Jemal, 2020), causing an enormous threat to human life and health. The occurrence of HCC is a complex process involving multiple factors and is affected by both genetics and the environment. Hepatitis B virus (HBV) and hepatitis C virus (HCV) infection, aflatoxin, alcohol abuse, and nitrosamines are all risk factors for the development of HCC (Villanueva, 2019). Early discovery is essential to improve survival rates and the quality of life of HCC patients, which requires us to explore novel HCC biomarkers. The unique role of circSMARCA5 in HCC suggests it can be used as a biomarker for the early diagnosis of HCC.

Several studies have reported that circSMARCA5 levels in HCC tissues are lower than those in matched normal tissues (Yu et al., 2018; Zhang et al., 2018; Li and Hu 2019). Moreover, the abundance of circSMARCA5 in plasma and tissues gradually decreases from healthy controls to hepatitis patients, cirrhosis patients and HCC patients, which indicates that circSMARCA5 expression is tissue-stage specific. Plasma circSMARCA5 is relatively stable under different temperature storage conditions, providing convenience for its clinical use as a marker (Li and Hu 2019).

However, in contrast to the downregulation of circSMARCA5, the levels of SMARCA5 mRNA and SMARCA5 proteins are all upregulated in HCC. With further study, it was found that circSMARCA5 in HCC was regulated posttranscriptionally by certain RBPs. Downregulation of circSMARCA5 is partly regulated by DExH-box helicase 9 (DHX9), an abundant nuclear RNA helicase. DHX9 inhibited the production of circSMARCA5 by binding to the reverse complementary sequence of circSMARCA5 and suppressing the pairing of these sequences (Yu et al., 2018). Functional experiments demonstrated that circSMARCA5 overexpression not only inhibited proliferation, migration, invasion and intrahepatic metastasis of HCC but also promoted late apoptosis (Zhang et al., 2018; Li and Hu 2019). Additionally, *in vivo* experiments revealed that circSMARCA5 overexpression suppressed the growth of subcutaneous xenograft tumors in nude mice and also resulted in a decrease in liver and lung metastasis. Mechanistically, circSMARCA5 increased the expression of TIMP3, which is a tumor suppressor that inhibits HCC progression, by acting as a sponge of miR-17-3p/miR-181b-5p (Yu et al., 2018). The clinicopathological characteristics of HCC patients, such as tumor differentiation, TNM stage, microvascular invasion, tumor size, and number of tumor lesions, were negatively correlated with circSMARCA5 abundance (Yu et al., 2018; Zhang et al., 2018; Li and Hu 2019).

ROC curves were constructed to assess the predictive value of circSMARCA5 in plasma for HCC, and the results showed that plasma circSMARCA5 could act as an ideal biomarker for distinguishing HCC cases from normal controls (AUC = 0.938), hepatitis cases (AUC = 0.853) and cirrhosis cases (AUC = 0.711). After combining it with α-fetoprotein (AFP), the AUC values were increased to 0.992, 0.903, and 0.858, respectively, which means that a combination of circSMARCA5 and AFP could significantly improve the diagnostic efficiency of HCC. Moreover, when AFP **<** 200 ng/ml, the AUCs of plasma circSMARCA5 for distinguishing HCC patients from hepatitis and cirrhosis patients were 0.847 and 0.706, respectively (Li and Hu 2019). Similar results were obtained from the ROC curve established by Zhang et al. (Zhang et al., 2018). In terms of prognosis, a low level of circSMARCA5 also predicted a shorter OS and recurrence-free survival (RFS) after surgery (Yu et al., 2018). In conclusion, circSMARCA5 plays an important role in suppressing the progression of HCC and is a potential diagnostic and prognostic biomarker for HCC patients.

### 7.4 CircSMARCA5 and non-small -cell lung cancer

Lung cancer is one of the cancers with the highest morbidity and mortality in humans (Sung et al., 2021). In 2020, more than 228,820 people were diagnosed with lung cancer in the United States (Siegel and Jemal, 2020). Smoking behavior is one of the most important factors in the occurrence of lung cancer. Non-small -cell lung cancer (NSCLC) accounts for approximately 85% of all lung cancer cases, and the overall cure rate and 5-year overall survival rate remain extremely low (Dumaand Molina 2019). To improve the therapeutic effect and prognosis of NSCLC, it is urgent to clarify the pathogenesis of NSCLC and find reliable early diagnostic biomarkers and therapeutic targets. It has been reported that circRNAs are involved in the occurrence and development of NSCLC and have diagnostic and prognostic potential for NSCLC (Wang et al., 2019).

Zhang et al. evaluated circSMARCA5 levels in 66 paired NSCLC tissues and adjacent tissues, and the results showed that the relative abundance of circSMARCA5 in NSCLC tissues was at a low level in contrast to that in normal tissues and was associated with patients’ clinical features, such as tumor size, lymphatic metastasis and TNM stage (Zhang et al., 2020). Similar results were obtained at the cellular level (Tong, 2020). CircSMARCA5 overexpression significantly inhibited the proliferation, migration, and invasion of NSCLC cells.

Meanwhile, chemotherapy sensitivity toward cisplatin and gemcitabine was also improved in circSMARCA5 overexpressing NSCLC cells (Tong, 2020). *In vivo* experiments also demonstrated that circSMARCA5 overexpression significantly diminished the tumor volume and tumor weight (Zhang et al., 2020), indicating a potential tumor inhibitory role of circSMARCA5 in NSCLC. Mechanistically, circSMARCA5, as a competing endogenous RNA of miR-670-5p, inhibited the progression of NSCLC by upregulating the expression levels of RBM24 (Zhang et al., 2020). In addition, in terms of prognosis, those patients with relatively high expression of circSMARCA5 exhibited better DFS and OS (Tong, 2020). In summary, circSMARCA5 is a tumor suppressor and might have value as a biomarker in NSCLC.

### 7.5 CircSMARCA5 and intrahepatic cholangiocarcinoma

Intrahepatic cholangiocarcinoma (ICC) is a rare malignant tumor of the digestive system with high mortality that accounts for approximately 10–15% of all primary hepatocellular carcinoma patients (Sirica et al., 2019; Sung et al., 2021). The incidence of ICC is increasing in the United States and is associated with several factors, such as hepatolithiasis, primary sclerosing cholangitis and *C. sinensis* (Uhlig et al., 2019). Surgical resection is currently the only treatment method effective for ICC, but its curability rate is extremely low (Zhang and Wu, 2016).

It has been reported that circRNAs are abnormally expressed in ICC, and can serve as potential molecular biomarkers for predicting tumor progression and poor outcomes of ICC patients (Jiang, 2018; Limb et al., 2020). Recently, circSMARCA5 was discovered to be abnormally expressed in ICC. Lu et al. demonstrated that the relative abundance of circSMARCA5 was reduced in 92 paired ICC tissues compared to adjacent normal tissues, which indicated that circSMARCA5 might function as a promising tumor inhibitory factor for ICC (Lu, 2020). Moreover, the clinical features of patients, including ECOG performance score, TNM stage and abnormal CA199 status, were all negatively associated with circSMARCA5 levels. The circSMARCA5 level acted as an independent predictive factor for the prognosis of ICC cases, and patients with high circSMARCA5 expression had a more favorable OS. Functional experiments indicated that circSMARCA5 overexpression could markedly inhibit cell proliferation and increase chemotherapy sensitivity of ICC cells to cisplatin or gemcitabine. In summary, circSMARCA5 might play significant antitumor roles in ICC.

### 7.6 CircSMARCA5 and multiple myeloma

Multiple myeloma (MM), characterized by malignant proliferation of plasma cells, is one of the most common hematological malignancies among adults (Legarda and de la Rubia 2020; Shah, 2020). In 2020, approximately 180,000 patients were newly diagnosed with MM, and 120,000 died of MM worldwide (Sung et al., 2021). A recent study revealed that circSMARCA5 might play a crucial role in the pathogenesis of MM. Liu et al. discovered that the expression of circSMARCA5 was dramatically decreased in MM patient bone marrow samples compared to healthy controls (Liu et al., 2019). Likewise, similar results could be observed in MM cell lines and normal plasma cells. CircSMARCA5 overexpression in MM cells not only reduced the proliferation of MM cells but also increased their apoptosis rate. Mechanistically, circSMARCA5 served as a sponge for miR-767-5p to inhibit MM cell proliferation and promote cell apoptosis. The ROC curves showed that circSMARCA5 could play an important role in distinguishing MM patients from controls, and its AUC value was 0.714. Beta-2-microglobulin is massively synthesized in MM patients, and patients with high circSMARCA5 expression have lower beta-2-microglobulin levels in their bodies. Furthermore, the patients who had more abundant circSMARCA5 exhibited higher rates of a complete response and longer OS and PFS after effective chemotherapy. In short, circSMARCA5 might act as an anti-oncogene in MM and could serve as a promising biomarker for the diagnosis and prognosis of MM patients.

### 7.7 CircSMARCA5 and colorectal cancer

Colorectal cancer (CRC), which is closely related to a poor diet and lifestyle, is the third most common malignant tumor worldwide (Li, 2016). More than 1,900,000 new diagnoses of CRC and 935,000 deaths occurred in 2020 (Sung et al., 2021). Genetics, lifestyle, obesity, and environmental factors all play a part in the etiology of CRC (Dekker et al., 2019). Recently, it has been reported that abnormally expressed circRNAs are related to the development of CRC (Artemaki and Konotos, 2020). Clarifying the specific roles and mechanisms of circRNAs in CRC is of great significance for guiding clinical diagnosis and treatment in the future.

Yang et al. revealed that the abundance of circSMARCA5 was dramatically reduced in CRC tissues compared to adjacent normal tissues (Yang Y et al., 2021). The level of circSMARCA5 in various CRC cells was also downregulated (Miao et al., 2020). Moreover, Functional experiments suggested that circSMARCA5 overexpression could clearly weaken CRC cell proliferation, migration and invasion and promote cell apoptosis (Miao et al., 2020; Yang G et al., 2021). Furthermore, circSMARCA5 overexpression in CRC cells promoted p53, p21, Bax and cleaved-Caspase-3 protein expression but inhibited CyclinD1 protein expression. Similarly, *in vivo* experiments also proved that circSMARCA5 upregulation inhibited the volume and weight of subcutaneously transplanted tumors in nude mice (Yang Y et al., 2021). Mechanistically, circSMARCA5 served as a competing endogenous RNA for miR-39-3p to upregulate the expression of ARID4B and inhibit CRC progression (Miao et al., 2020) and circSMARCA5 also sponged miR-552 to inhibit CRC progression by inactivating the Wnt and YAP1 pathways (Yang G et al., 2021). In another study, researchers detected the expression of circSMARCA5 in the plasma of colorectal cancer patients. Kaplan-Meier analysis showed that patients with low circSMARCA5 expression in the plasma had a shorter OS (Radanova et al., 2021). In brief, circSMARCA5 may function as a prognostic marker and as an antitumor candidate to inhibit the progression of CRC.

### 7.8 CircSMARCA5 and cervical cancer

Cervical cancer (CC) is the fourth most common malignant tumor in women worldwide and is closely associated with human papillomavirus (HPV) infection (Small et al., 2017; Cohen and Oaknin 2019). Approximately 604,000 women were diagnosed with CC and 342,000 women died of CC in 2020 (Sung et al., 2021). HPV infection is an important cause of CC, and the treatment of CC is still a very difficult problem. However, recent studies found that circRNAs might be a potential therapeutic target for CC (Shi et al., 2020).

Tian et al. discovered that the circSMARCA5 level was downregulated in CC tissues compared to matched adjacent tissues (Tian, 2018). The same results also appeared in CC cell lines and normal cell lines (Zhang X et al., 2021). Furthermore, circSMARCA5 levels were gradually decreased in cervical intraepithelial neoplasia, stage I-II CC and stage III-IV CC. Functional experiments indicated that circSMARCA5 overexpression obviously suppressed CC cell proliferation, migration and invasion and enhanced cell apoptosis (Tian, 2018; Zhang Z et al., 2021). Moreover, the CC cell cycle distribution was also affected by circSMARCA5; upregulated circSMARCA5 significantly increased the proportion of the cells in G1 phase, while that in S phase was significantly reduced. Mechanistically, on the one hand, circSMARCA5 suppressed CC progression by serving as a sponge of miR-620. On the other hand, circSMARCA5 interacted with the RBP SND1 (an oncoprotein) and prevented the binding of SND and YWHAB, thereby inhibiting the proliferation and invasion of CC (Zhang X et al., 2021). Generally, circSMARCA5 is a promising therapeutic target for cervical cancer.

### 7.9 CircSMARCA5 and breast cancer

At present, breast cancer (BC) has become the most common malignant tumor in the world. In 2020, approximately 2,261,400 patients were diagnosed with BC and 684,996 deaths occurred worldwide (Sung et al., 2021). There are various treatments for BC, such as surgery, chemotherapy, radiotherapy, endocrine therapy and targeted therapy (Harbeck, 2017). In recent years, the therapeutic effect of circRNAs in BC has been extensively studied, showing that the emergence of circRNAs is expected to provide novel targets for BC therapy (Li et al., 2019).

Xu et al. discovered that circSMARCA5 levels were markedly reduced in BC tissues and cells compared to paracancerous tissues and immortalized breast epithelial cells (Xu et al., 2020). Additionally, the ratio of circSMARCA5 to linear SMARCA5 in healthy volunteer blood was also markedly higher than that in BC patients. Subsequently, the effects of circSMARAC5 on its parental gene SMARCA5 were investigated, and the results suggested that circSMARCA5 overexpression markedly decreased the expression of SMARCA5 mRNA and proteins. Conversely, knockdown of circSMARCA5 increased the expression of SMARCA5. Mechanistically, circSMARCA5 can bind exons 15–16 of SMARCA5 genomic DNA to form R-loops, which terminate SMARCA5 gene transcription and decrease the expression level of SMARCA5 in cancer cells. The low expression of SMARCA5 means the cells are unable to effectively repair DNA damage and maintain genome stability, resulting in increased sensitivity of BC to cisplatin or bleomycin. In short, overexpression of circSMARCA5 can enhance the chemosensitivity of BC cells, and circSMARCA5 might be a promising therapeutic target for BC drug-resistant patients.

### 7.10 CircSMARCA5 and prostate cancer

Prostate cancer (PCa) is one of the most commonly diagnosed cancers among men worldwide, and approximately 1,414,259 new cases of PCa were diagnosed in 2020 (Sung et al., 2021). Early diagnosis is critical to the prognosis of PCa patients. Therefore, massive efforts should be made to identify new reliable biomarkers of PCa. Fortunately, the unique characteristics of circRNAs make them ideal noninvasive diagnostic biomarkers for PCa detection (Fang and Dong, 2020).

It was reported that the abundance of circSMARCA5 was significantly increased in PCa tissues compared to matched normal prostate tissues (Kong et al., 2017; Dong et al., 2021), with similar results at the cellular level. CircSMARCA5 acted as an androgen-responsive gene, and the expression of circSMARCA5 in PCa could be induced by DHT in a dose-dependent manner; it was also significantly decreased after silencing the androgen receptor (Kong et al., 2017). A series of loss-of-function assays showed that knockdown of circSMARCA5 suppressed proliferation, migration, invasion, glycolysis and metastasis in PCa cell lines. Moreover, silencing of circSMARCA5 promoted cell apoptosis and inhibited cell cycle progression, with an increase in the proportion of cells in G1 phase and a reduction in the proportion of cells in S phase (Kong et al., 2017; Dong et al., 2021). Mechanistically, circSMARCA5 accelerated the progression of PCa by modulating the circSMARCA5/miR-432/PDCD10 signaling pathway. *In vivo* experiments also showed that circSMARCA5 knockdown suppressed PCa tumor growth in nude mice (Dong et al., 2021). In summary, circSMARCA5 functions as an oncogene for PCa and might be useful as a promising biomarker of PCa.

### 7.11 CircSMARCA5 and bladder cancer

Bladder cancer (BCa) is a common malignant tumor of the genitourinary system (Lenis and Chamie, 2020). In 2020, there were 573,278 new cases of BCa and 212,536 deaths worldwide (Sung et al., 2021), and the incidence and mortality of BCa has increased year by year, posing a great threat to human life and health. The occurrence and development of BCa is a multistage, slow-progressing and multifactorial pathological process. Numerous studies have suggested that circRNAs are associated with the occurrence and progression of BCa (Yang Y et al., 2021) and they are expected to be diagnostic and therapeutic markers for BCa. Zhang et al. (Zhang Z et al., 2021) detected circSMARCA5 levels in 156 BCa tissues and 71 paracancerous tissues, finding that circSMARCA5 was elevated in BCa tissues. In addition, analysis of the clinicopathological features demonstrated that patients with high circSMARCA5 expression tended to exhibit a larger tumor size, higher tumor stage and lymphatic metastasis. Similarly, the level of circSMARCA5 in several BCa cell lines was also upregulated (Tan and Liang, 2019). *In vitro* experiments illustrated that knockdown of circSMARCA5 obviously suppressed BCa cell proliferation, migration and invasion, and the apoptosis rate was significantly increased (Tan and Liang, 2019). At the same time, the expression of apoptosis-related proteins such as caspase-3 was increased, and the level of bcl-2 protein was decreased. In terms of prognosis, the expression level of circSMARCA5 was an independent predictive factor of DFS and OS in BCa patients, and patients with high circSMARCA5 expression had worse DFS and OS (Zhang Z et al., 2021).

## 8 Meta-analysis

### 8.1 Methods

Methods of meta-analysis were described in supplementary material.

### 8.2 Characteristics of the enrolled studies

**FIGURE 8:**
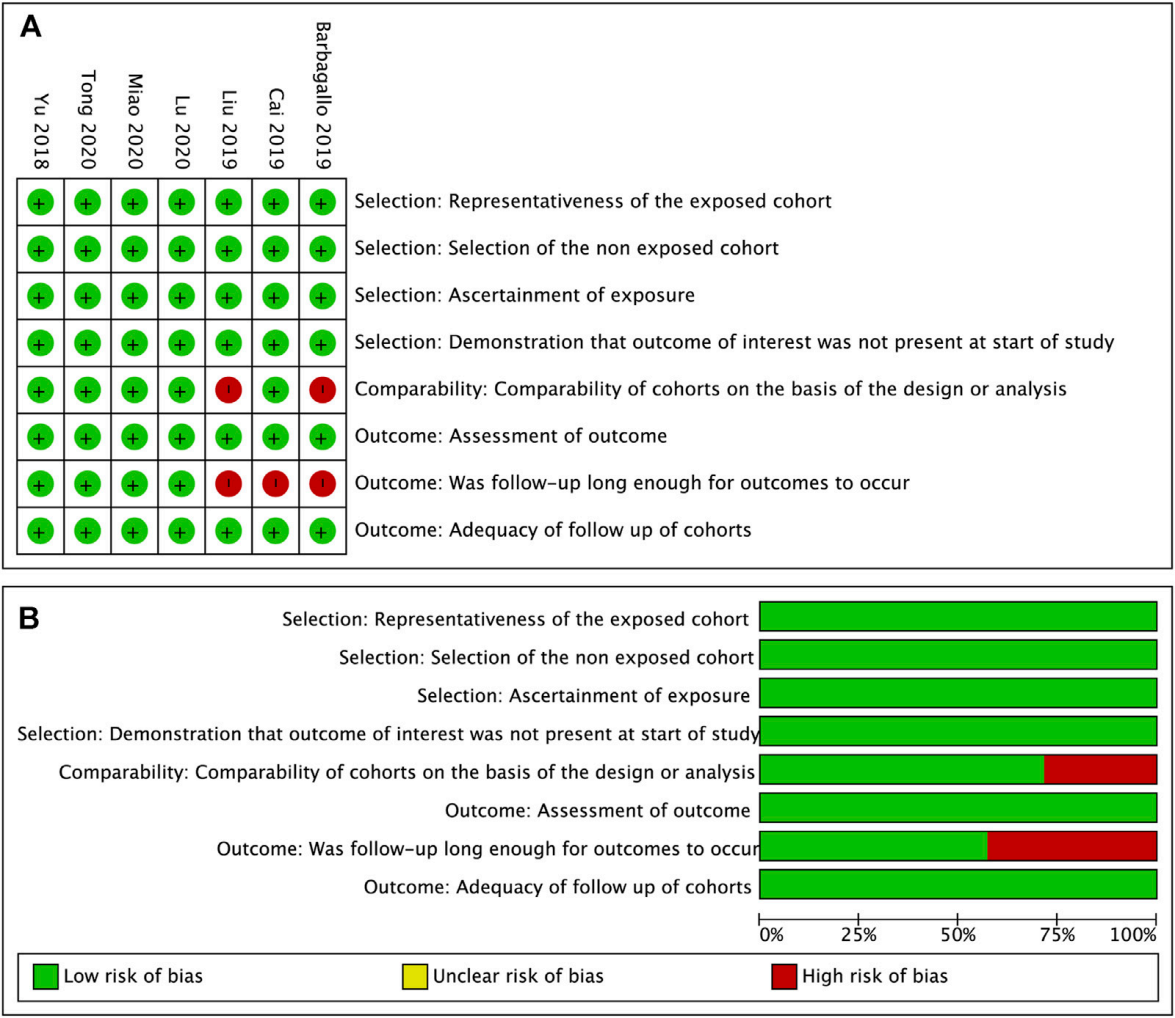
Quality assessment of the included studies. **(A)**. Each risk of bias item for each included study. **(B)**. Each risk of bias item presented as percentages across all included studies.

Seven enrolled studies were published from 2018 to 2020 and 7 different tumor types were involved, including GBM, GC, MM, NSCLC, ICC, CRC and HCC. The expression of circSMARCA5 was detected by qRT-PCR in all included studies. The characteristics of 7 studies are listed in Table 3. The quality of included studies was assessed by NOS quality evaluation scale. The methodological quality of most included studies was high (Figure 8).

**TABLE 3:** Characteristics of the studies included in the present meta-analysis.

Study	year	Cancer	No.	Detection method	Survival Outcome	Follow-up time (month)	NOS score
Barbagallo	2019	GBM	31	RT-qPCR	OS, PFS	≥40	6
Cai	2019	GC	60	RT-qPCR	OS, DFS, TNM	≥40	7
Liu	2019	MM	105	RT-qPCR	OS, PFS	≥40	6
Lu	2020	ICC	92	RT-qPCR	OS, TNM	≥60	8
Miao	2020	CRC	45	RT-qPCR	OS	≥100	8
Tong	2020	NSCLC	460	RT-qPCR	OS, DFS, TNM	≥96	8
Yu	2018	HCC	40	RT-qPCR	OS, RFS	≥60	8

Abbreviations: GC: gastric cancer; GBM: glioblastoma; HCC: hepatocellular carcinoma; NSCLC: non-small -cell lung cancer; ICC: intrahepatic cholangiocarcinoma; MM: multiple myeloma; CRC: colorectal cancer.

### 8.3 Association between circSMARCA5 expression and prognosis of patients

**FIGURE 9:**
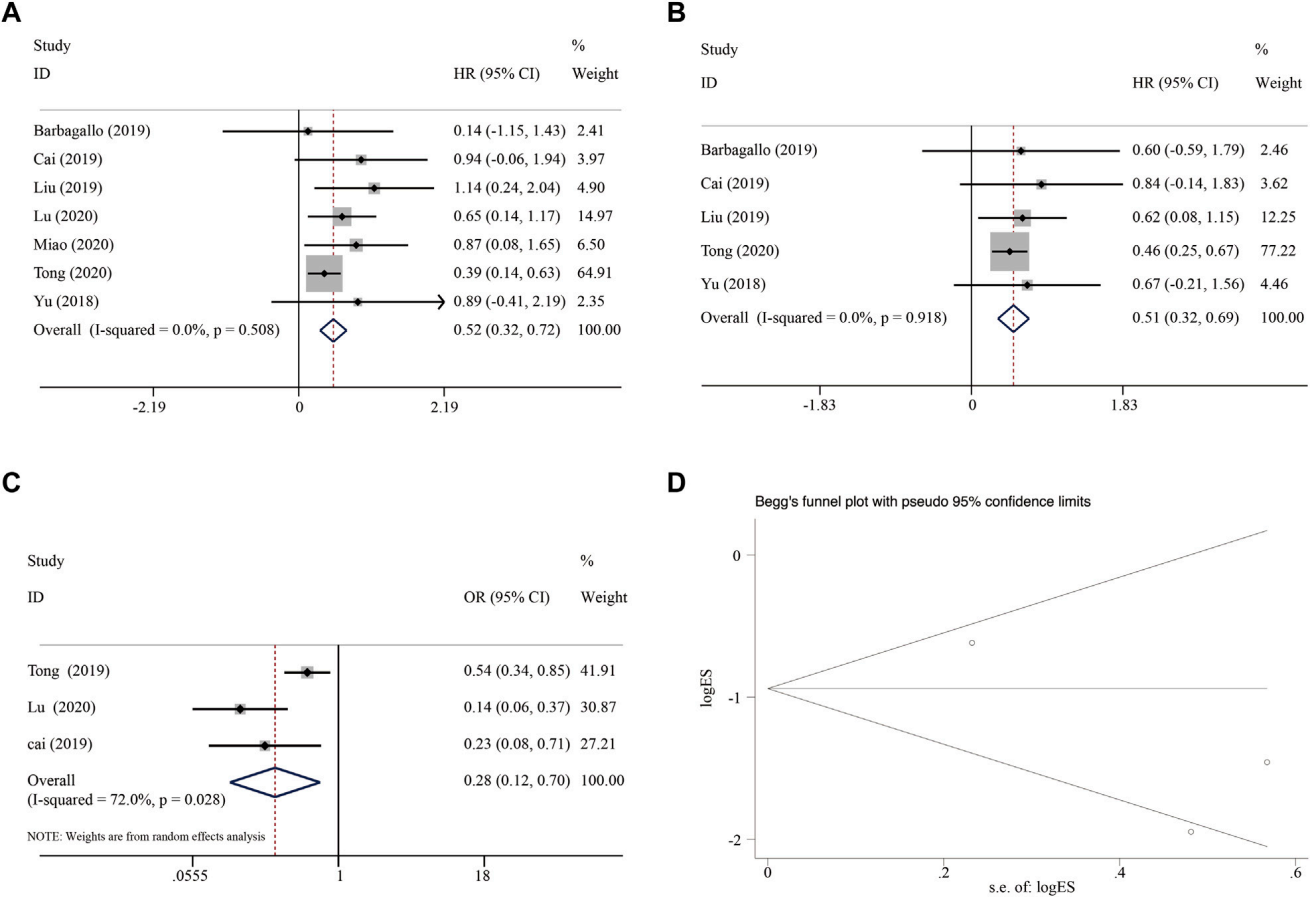
Forest plot of the association between circSMARCA5 expression and **(A)** OS, **(B)** DFS/RFS/PFS, **(C)** tumor TNM stage. **(D)** Begg’s funnel plot.

Meta-analysis of the 7 included studies showed that patients with high expression of circSMARCA5 exhibited better OS (HR = 0.52, 95% CI 0.32–0.72, I-squared = 0.0%, *p*-value = 0.508) (Figure 9A). Furthermore, the expression of circSMARCA5 in tumors was positively associated with DFS/RFS/PFS in patients (HR = 0.51, 95% CI 0.32–0.69, I-squared = 0.0%, *p*-value = 0.918) (Figure 9B). To further clarify the relationship between the expression of circSMARCA5 and tumor TNM stage, the meta-analysis of 3 eligible studies involving 612 patients showed that patients with high circSMARCA5 expression behaved less advanced TNM stage than those with low expression (HR = 0.28, 95% CI 0.12–0.70, I-squared = 72.0%, *p*-value = 0.028) (Figure 9C). The potential publication bias was estimated by Begg’s funnel plot. As shown in Figure 9D, the Begg’s funnel plot is symmetric and the *p*-value is 1.00, we judged that there was no publication bias.

## 9 CircSMARCA5 and other diseases

### 9.1 CircSMARCA5 and osteoporosis

Osteoporosis (OPO), which is closely associated with a decline in serum estrogen, is a common chronic bone metabolic disease in postmenopausal women (Li, 2018). The characteristics of OPO are reduced bone mass and microarchitectural deterioration of bone tissue, leading to many adverse bone events (Compston and Leslie 2019). Many circRNAs have been found to promote osteogenic differentiation in OPO (Yu, 2019; Lin et al., 2020; Wen W et al., 2020). Recently, Xiang et al. (Xiang, 2020) reported that circSMARCA5 was involved in the progression of OPO. The expression of circSMARCA5 was lower in the plasma of OPO patients than in that of osteopenia patients and healthy controls. β-Isomerized C-terminal telopeptides (β-CTx), an indicator of bone resorption, were negatively correlated with the abundance of circSMARCA5, and the results indicated that circSMARCA5 might reflect a reduction in bone resorption and an increase in bone formation. Moreover, ROC curve analysis was performed to assess the diagnostic value of circSMARCA5, and the results showed that the levels of circSMARCA5 in plasma could distinguish OPO patients from healthy controls. The sensitivity and specificity were 0.976 and 0.822, respectively, and the AUC value was 0.9589, which meant that circSMARCA5 was a potential diagnostic biomarker. After OPO patients received effective anti-osteoporotic treatment for 6 months, the patients’ bone mass was increased and their plasma level of circSMARCA5 was also significantly upregulated compared to pretreatment. In summary, circSMARCA5 could act as a diagnostic indicator for OPO patients and is a promising treatment target.

### 9.2 CircSMARCA5 and cardiovascular diseases

Cardiovascular diseases are one of the major causes of noninfectious deaths among middle-aged and elderly people. Atherosclerosis (AS) is the pathological basis of many related cardiovascular diseases (Shah, 2019). Among them, vascular inflammation has an important influence on the various processes of AS (Zhu et al., 2018; Ruparelia, 2020). It has been reported that the expression of circSMARCA5 is downregulated in AS (Liang and Xin, 2021).

Human umbilical vein endothelial cells (HUVECs) treated with oxidized low-density lipoprotein (oxLDL) are used to simulate AS *in vitro*. Studies have demonstrated that the expression of circSMARCA5 is decreased in oxLDL-treated HUVECs in a dose-dependent manner (Cai et al., 2020; Liang and Xin, 2021). Nevertheless, circSMARCA5 overexpression reversed the inhibition of proliferation, angiogenesis, apoptosis, and migration of oxLDL-treated HUVECs *in vitro*. Moreover, overexpression of circSMARCA5 obviously alleviated the inflammatory response of oxLDL-treated HUVECs, and the expression of inflammatory factors, such as TNF-α, IL-6 and IL-1β, was decreased in oxLDL-treated HUVECs. Mechanistically, studies have shown that serine/arginine-rich splicing factors (SRSF) are a risk factor for coronary heart disease (Tejedor et al., 2015). CircSMARCA5 overexpression could reverse the oxLDL-induced HVUEC proliferation inhibition by activating the SRSF1/β-catenin axis (Liang and Xin, 2021). CircSMARCA5 also reduced the inflammatory response and oxidative stress of oxLDL-treated HUVECs by acting as a competitive endogenous RNA for miR-640 (Cai et al., 2020). Another study demonstrated that circSMARCA5 was highly stable in plasma samples and that the expression of circSMARCA5 was negatively correlated with the severity and extension of coronary heart disease (Liang and Xin, 2021). Overall, circSMARCA5 is a valuable diagnostic biomarker and a promising therapeutic target in cardiovascular diseases.

## 10 Conclusion and prospect

Accumulating evidence suggests that circRNAs act as tumor suppressor or play oncogenic roles in the occurrence and development of cancer, and they also have great potential as diagnostic and prognostic biomarkers in tumors in the future. In this review, we summarize the role of circSMARCA5 in various diseases. CircSMARCA5 functions as a tumor suppressor in most cancers, and the expression of circSMARCA5 is negatively associated with clinicopathological features such as tumor size, TNM stage, lymphatic metastasis and vascular invasion, indicating that circSMARCA5 will be of great value in clinical applications in the future. Furthermore, a meta-analysis of 7 enrolled studies about circSMARCA5 showed that the expression of circSMARCA5 was positively correlated with prognosis of patients. However, a few studies showed that circSMARCA5 plays a tumor-promoting role in cervical cancer, prostate cancer and bladder cancer (Huang Q et al., 2020; Dong et al., 2021; Zhang Z et al., 2021). In terms of mechanism, circSMARCA5 mainly serves as a miRNA sponge in various cancers. CircSMARCA5 can also bind to various RNA-binding proteins such as SRSF1 and SND1 to play a tumor-suppressing role in glioblastoma and cervical cancer (Barbagallo et al., 2019; Zhang X et al., 2021). In addition, the sequence of circSMARCA5 is predicted to have ORFs, IRESs and m6A motifs, indicating that circSMARCA5 has the potential to code for proteins or functional peptides. To date, research on circSMARCA5 is far from sufficient. Past studies mainly focused on the role of circSMARCA5 in tumors. However, research on circSMARCA5 in nonneoplastic diseases such as osteoporosis and cardiovascular disease is still scarce. With more research to carry out on circSMARCA5 in other diseases, our understanding of the pathogenic mechanism of circSMARCA5 will become more complete. Furthermore, with the advent of technology of synthetic circRNAs *in vitro* (Lu et al., 2022; Qu et al., 2022), synthetic drugs targeted circSMARCA5 may become a therapeutic option for some cancers in the future.
