# Supplementary material for: CircSMARCA5: A key circular RNA in various human diseases
Source: Front Genet. 2022 Aug 23;13:921306. doi: 10.3389/fgene.2022.921306 (PMC9445203; doi:10.3389/fgene.2022.921306)
Supplement: Supplementary file 1 [file DataSheet1.DOCX]

**Methods**

**Search Strategy**

To obtain eligible studies, we searched for the publications in the databases including PubMed, Web of Science, Embase, China National Knowledge Infrastructure (CNKI). The publications searched were updated to June 2022. We searched for studies related to circSMARCA5 using the following keywords: circSMARCA5, circular RNA SMARCA5, circular RNA cSMARCA5 or hsa_circ_0001445.

**Inclusion and Exclusion Criteria**

The inclusion criteria were as follows: (1) the expression level of circSMARCA5 was detected in various human cancers; (2) Studies in which patients were stratified by the expression levels of circSMARCA5; (3) The association between circSMARCA5 expression and patient prognosis and tumor TNM stage was described in publications. The exclusion criteria were as follows: (1) duplicate publications; (2) review, comment, conference abstract and letter; (3) Studies not related to circSMARCA5.

**Data Extraction**

Liu Hongwei and Huang Gaozhen extracted the data from the identified publications independently. The following information was extracted from each publication: first author, publication year, cancer type, number of patients in low expression and high expression groups, detection method, survival outcome, follow-up time.

**Quality Assessment**

The quality assessment of diagnostic accuracy studies Newcastle-Ottawa Score (NOS) quality assessment system^[1]^ was used to assess the quality of enrolled studies. Enrolled studies were scored based on case definition, representation of cases, selection restrictions, definition of controls, comparability of cases and controls, determination of exposure, identical determination methods for cases and controls, and nonresponse rates. Studies with a score≥6 were considered high quality.

**Statistical Analysis**

Results were visualized using STATA 17SE software and Review Manager 5.3 software. The heterogeneity test was performed by I-squared statistics. We analyzed the data using a fixed-effects model by default and switched to a random-effects model if I-squared > 50%. We judged that there was significant heterogeneity among the included studies when the P-value was <0.05, otherwise, there was no significant heterogeneity.

For the prognostic meta-analysis, pooled RR and 95% CI was used to describe the prognostic value of circSMARCA5 expression. The potential publication bias was estimated by using Begg’s funnel plot. We judged that there was no publication bias if P-value>0.1 for Begg’s test.

[1] STANG A. Critical evaluation of the Newcastle-Ottawa scale for the assessment of the quality of nonrandomized studies in meta-analyses. Eur J Epidemiol, 2010, 25(9): 603-5.
